# Datasets of mass of phosphorus flows in Zhangzhou city in China

**DOI:** 10.1016/j.dib.2019.104080

**Published:** 2019-05-29

**Authors:** Chu-Long Huang, Shenghui Cui

**Affiliations:** a Key Lab of Urban Environment and Health, Institute of Urban Environment, Chinese Academy of Sciences, 1799, Jimei Road, Xiamen 361021, China; b Department of Resources and Environmental Sciences, Quanzhou Normal University, 398, Donghai Street, Quanzhou 362000, China; c Department of Civil and Environmental Engineering, University of Michigan, Ann Arbor, MI 48109-2125, United States

## Abstract

Compared to currency data which tends to fluctuate with the market and cannot accurately reflect the effects of human activities on resources use efficiency and environmental sustainability, the assessment accuracy of the effects can be further improved by constant mass data of elements contained in materials and products within socioeconomic system, e.g. phosphorus mass data for its use efficiency assessment in the case of Zhangzhou city in China Huang et al., 2019. Firstly, the mass data of products and raw materials were sourced or assessed mainly from government statistical year books or bulletins. Secondly, the phosphorus contents in materials were derived mainly from literature. Finally, the mass of phosphorus flows throughout food production-consumption system in Zhangzhou prefecture, Fujian province, China was accounted by mass balance accounting based on substance flow analysis. These data include the following parts: input/output phosphorus flows across the jurisdiction boundary of Zhangzhou city; phosphorus flows into or out of agricultural production subsystems such as crop farming, livestock, and aquaculture; phosphorus flows into or out of human consumption subsystem; phosphorus flows across subsystems. Part of reference data related to phosphorus mass balance accounting was also presented.

**Table:** Specifications table

Subject area	*Environmental management.*
More specific subject area	*Environmental system analysis, element metabolism in socioeconomic system.*
Type of data	*Table.*
How data was acquired	*Survey by local governmental agencies, literature, reports, yearbooks, etc.; Mass balance modeling by substance flow analysis.*
Data format	*Raw, filtered, analyzed, extrapolated.*
Experimental factors	The mass of phosphorus flows throughout food production-consumption system in Zhangzhou prefecture were assessed.
Experimental features	*Mass balance thinking and bottom-up assessment method were applied.*
Data source location	*Yearbooks, reports, interviews across the jurisdiction of Zhangzhou City in China and global literature.*
Data accessibility	*The phosphorus mass data is with this article.*
Related research article	**[1]****Huang, C.L.**, Gao, B., Xu, S., Huang, Y.F., Yan, X.M., Cui, S.H.,2019.Changing phosphorus metabolism of a global aquaculture city. Journal of Cleaner Production. 225: p.1118–1133*.”*

**Table:** 

**Value of the data** • Mass data of phosphorus element flows across regional socioeconomic system can be an integrated part of global map of element biogeochemical cycle. • Mass data within socioeconomic system is more valuable and convincing for global sustainability assessment, compared to currency data. • Analysis of systematic data derived from substance flow analysis of element flows in the interaction between human and the environment will provide viable approach for getting a solution for the dilemma between economic development and environmental protection. • Compared with other element data in this city or the same element in other cities, effects of urbanization or industrial structure on element metabolism efficiencies can be verified.

## 1 Data

The datasets of this article provide information on the phosphorus use of agricultural food production subsystems and urban/rural food consumption subsystems and the phosphorus emission from these subsystems. Table 1, Table 2, Table 3, Table 4 showed the input/output phosphorus flows of each subsystem. Table 5 showed the import/export phosphorus flows across the boundary of the whole system.

**Table 1:** Phosphorus flows for crop farming subsystems Unit: tonnes.

Year	Fertilizers	Pesticides	Seeds	FP	F	TS	RS	Stubble	RM	RRE	RUE
2014	33023	194	55	12193	212	1123	178	321	3818	863	144
2013	31090	196	56	11585	214	1135	186	315	4532	856	147
2012	31673	193	55	11095	215	1114	187	308	4577	849	149
2011	31782	198	56	10793	222	1099	187	301	4582	848	142
2010	31874	198	56	10441	223	1124	196	299	4231	848	141
2009	31590	195	55	10236	228	1132	200	295	3820	837	143
2008	29434	190	51	10270	240	1139	205	295	3797	850	137
2007	28664	178	47	9721	252	1208	218	298	3602	844	134
2006	29544	182	50	9434	266	1222	223	300	3583	844	128
2005	30373	185	73	10883	267	1474	281	362	4502	841	119
2004	30028	172	74	10753	253	1526	291	371	4588	840	118
2003	29393	177	75	10427	274	1591	306	372	4366	842	105
2002	29133	175	78	10337	263	1598	307	374	4343	802	120
2001	29188	178	87	11147	291	1681	337	389	4443	995	72
2000	32232	147	96	11280	290	1669	342	388	4359	1029	76
1999	33825	165	113	11686	282	2178	471	442	4275	1041	75
1998	31473	142	116	11715	296	2697	594	498	4375	1052	79
1997	30739	1218	119	11335	285	2729	612	491	4177	1084	82
1996	28267	686	120	11125	317	2674	599	480	4236	1100	92
1995	25788	153	117	10401	310	2557	582	448	4036	1113	98

Notes: FP, food products from crop farming; F, green fodder; TS, total straw output; RS, recycled straw; RM, recycled livestock manure applied to the field; RRE/RUE, recycled rural/urban residents' excreta applied to the field.

**Table 2:** Phosphorus flows for livestock subsystems Unit: tonnes.

Year	IFL	S	F	G	LP	LL	RM
2014	3734	317	212	935	588	793	3818
2013	4638	310	214	927	674	883	4532
2012	4653	304	215	931	665	862	4577
2011	4538	298	222	914	640	750	4582
2010	4270	294	223	896	611	842	4231
2009	3991	288	228	865	586	967	3820
2008	3914	286	240	841	573	910	3797
2007	3564	284	252	760	559	700	3602
2006	3785	284	266	749	579	922	3583
2005	4427	347	267	1157	748	948	4502
2004	4476	354	253	1152	755	893	4588
2003	4128	346	274	1085	730	737	4366
2002	4066	346	263	1080	715	697	4343
2001	3877	376	291	1285	722	663	4443
2000	3653	381	290	1378	710	634	4359
1999	3029	425	282	1574	647	388	4275
1998	3011	458	296	1635	648	376	4375
1997	3340	451	285	1023	594	328	4177
1996	2573	442	317	1558	540	114	4236
1995	2342	411	310	1457	474	9	4036

Notes: IFL, imported feed for livestock; S, straw feed; F, green fodder; G, grain feed; LP, livestock products; LL, Loss from livestock; RM, recycled livestock manure applied to the field.

**Table 3:** Phosphorus flows for aquaculture subsystems Unit: tonnes.

Year	IFSF	IFFF	MFP	APU	APR	LSA	LFA	FAP	SP	SAP	SAPF
2014	3358	29882	5236	103	179	2567	27733	2149	6028	3579	791
2013	3016	28180	4965	92	158	2282	26153	2027	5698	3349	733
2012	2874	26896	4773	83	144	2173	24961	1935	5473	3170	701
2011	2423	25283	4696	77	140	1810	23465	1819	5310	3053	613
2010	2282	24116	4577	65	143	1696	22381	1735	5164	2945	587
2009	2301	23473	4493	72	153	1716	21784	1688	5077	2891	585
2008	2188	22535	4410	62	139	1641	20914	1621	4957	2809	547
2007	2041	21074	4399	75	131	1583	19558	1516	4857	2738	458
2006	1902	19431	4327	65	122	1475	18034	1398	4754	2681	427
2005	2165	19439	4909	66	122	1660	18040	1398	5414	3073	505
2004	2162	19516	4737	72	113	1665	18112	1404	5234	2918	497
2003	1968	17138	4645	59	104	1570	15905	1233	5043	2791	398
2002	2047	17266	4407	63	99	1536	16024	1242	4918	2696	511
2001	1909	14821	4486	35	116	1526	13755	1066	4869	2633	383
2000	1490	13467	4468	32	101	1184	12498	969	4774	2549	306
1999	991	12391	4180	26	94	794	11500	891	4378	2260	197
1998	1131	11313	3874	21	91	932	10499	814	4074	1978	200
1997	345	10539	3591	16	87	282	9781	758	3654	1762	63
1996	396	8645	1912	11	91	278	8023	622	2030	632	118
1995	415	7401	1688	6	89	259	6868	532	1844	502	156

Notes: IFSF/IFFF, imported feed for seawater/freshwater fauna; MFP, marine fishing products; APR/APU, aquaculture products for rural/urban residents; LFA/LSA, loss from freshwater/seawater aquaculture; LP, livestock products; FAP, freshwater aquaculture products; SP, seawater products; SAP, seawater aquaculture products; SAPF, seawater aquaculture products on feed.

**Table 4:** Phosphorus flows for human consumption subsystems Unit: tonnes.

Year	CU	CR	LPR	LPU	APU	APR	SF	RRE	RUE	LR	LU	UFI
2014	446	1040	378	247	103	179	126	863	144	1252	703	480
2013	370	1067	349	273	92	158	135	856	147	1226	716	500
2012	383	1117	358	306	83	144	129	849	149	1200	729	407
2011	343	1140	345	275	77	140	129	848	142	1184	717	442
2010	326	1149	289	249	65	143	141	848	141	1169	715	512
2009	328	1087	273	248	72	153	150	837	143	1139	749	556
2008	294	1114	259	213	62	139	154	850	137	1143	720	614
2007	352	1120	266	278	75	131	189	844	134	1120	733	420
2006	324	1094	269	285	65	122	194	844	128	1120	727	466
2005	357	1124	271	282	66	122	213	841	119	1118	730	374
2004	378	1271	245	283	72	113	227	840	118	1115	723	207
2003	294	1344	239	274	59	104	265	842	105	1118	706	192
2002	370	1402	227	285	63	99	268	802	120	1066	770	45
2001	242	1812	249	120	35	116	255	995	72	1240	441	(80)
2000	232	1524	228	123	32	101	236	1029	76	1200	431	260
1999	216	1490	212	115	26	94	387	1041	75	1176	395	147
1998	200	1458	204	106	21	91	562	1052	79	1150	387	28
1997	184	1427	196	97	16	87	575	1084	82	1112	372	68
1996	168	1428	196	88	11	91	565	1100	92	1092	347	82
1995	152	1428	196	79	6	89	547	1113	98	1069	329	112

Notes: CR/CU, crop products for rural/urban residents; LPR/LPU, livestock products consumed by rural/urban residents; APU/APR, aquaculture products for urban/rural residents; SF, straw fuel; RRE/RUE, recycled rural/urban residents' excreta applied to the field; LR/LU, loss from rural/urban residents' consumption; UFI, Unknown food inflow.

**Table 5:** The import/export phosphorus flows across the boundary of the whole system Unit: tonnes.

Year	Import flows						Export flows			Total export	Total import
	Fertilizers	Pesticides	Seeds	IFSF	IFFF	IFL	EC	EL	EA		
2014	33023	194	55	3358	29882	3734	9771	0	7895	17629	70246
2013	31090	196	56	3016	28180	4638	9221	52	7475	16748	67175
2012	31673	193	55	2874	26896	4653	8665	0	7181	15846	66344
2011	31782	198	56	2423	25283	4538	8396	19	6912	15327	64281
2010	31874	198	56	2282	24116	4270	8069	73	6691	14834	62797
2009	31590	195	55	2301	23473	3991	7957	64	6541	14561	61604
2008	29434	190	51	2188	22535	3914	8022	101	6377	14499	58311
2007	28664	178	47	2041	21074	3564	7489	14	6168	13670	55569
2006	29544	182	50	1902	19431	3785	7268	24	5965	13257	54894
2005	30373	185	73	2165	19439	4427	8245	196	6625	15065	56661
2004	30028	172	74	2162	19516	4476	7951	228	6453	14631	56427
2003	29393	177	75	1968	17138	4128	7704	217	6112	14033	52879
2002	29133	175	78	2047	17266	4066	7485	203	5998	13687	52766
2001	29188	178	87	1909	14821	3877	7809	354	5784	13947	50060
2000	32232	147	96	1490	13467	3653	8146	358	5610	14114	51084
1999	33825	165	113	991	12391	3029	8405	320	5148	13874	50513
1998	31473	142	116	1131	11313	3011	8422	339	4775	13536	47185
1997	30739	1218	119	345	10539	3340	8701	302	4309	13312	46301
1996	28267	686	120	396	8645	2573	7971	256	2550	10777	40688
1995	25788	153	117	415	7401	2342	7365	199	2281	9845	36217

Notes: IFSF/IFFF, imported feed for seawater/freshwater fauna; IFL, imported feed for livestock; EC, exported crop products; EL, exported livestock products; EA, exported aquaculture products.

These datasets were based on the use of phosphorus-containing products, including products for the purposes of production or consumption, and the phosphorus content in the products. The detailed calculation methods can be seen in the Appendix A. Supplementary data for Huang et al. (2019) [1]. Based on these datasets, phosphorus flow chart for each year can be drawn, e.g. the year 1995 and 2014 in Huang et al. (2019) [1].

## 2 Experimental design, materials, and methods

### 2.1 Analytical framework and data sources

We applied a coupled system approach, i.e. Substance Flow Analysis (SFA), to account for the phosphorus flows through regional food production and consumption system and its subsystems. Analytical framework and data sources can be seen in Huang et al. (2019) [1].

### 2.2 Mass balance modeling

The equations of all phosphorus inputs and outputs during production, consumption, and discharge were constructed based on the mass balance principle, see Huang et al. (2019) [1].
